# Evaluation of Zhenwu Decoction Effects on CYP450 Enzymes in Rats Using a Cocktail Method by UPLC-MS/MS

**DOI:** 10.1155/2020/4816209

**Published:** 2020-05-12

**Authors:** Li-li Hong, Qian Wang, Ya-ting Zhao, Sheng Zhang, Kai-qi Zhang, Wei-dong Chen, Can Peng, Li Liu, Hong-song Wang

**Affiliations:** ^1^School of Pharmacy, Anhui University of Chinese Medicine, Hefei, Anhui 230012, China; ^2^Anhui Province Key Laboratory of Chinese Medicinal Formula, Hefei, Anhui 230012, China; ^3^Synergetic Innovation Center of Anhui Authentic Chinese Medicine Quality Improvement, Hefei, Anhui 230012, China; ^4^Institute of Pharmaceutics, Anhui Academy of Chinese Medicine, Hefei, Anhui 230012, China; ^5^School of Pharmacy, China Pharmaceutical University, Nanjing 211198, China; ^6^Institute of Traditional Chinese Medicine, Anhui University of Chinese Medicine, Hefei, Anhui 230012, China

## Abstract

This thesis is aimed at shedding light on the effects of the Zhenwu decoction (ZWD) on the activities and mRNA expressions of seven CYP450 isoenzymes. In the first step, we determined the main chemical compounds of ZWD by high-performance liquid chromatography (HPLC). Next, 48 male (SD) rats were randomly divided into the normal saline (NS) group and the ZWD low- (2.1875 g/kg), medium- (4.375 g/kg), and high- (8.75 g/kg) dose groups (12 per group). All rats were gavaged once daily for 28 consecutive days. A mixed solution of seven probe drugs was injected into 24 rats through the caudal vein after the last intragastric administration. Lastly, a validated cocktail method and real-time quantitative reverse-transcription polymerase chain reaction (RT-qPCR) were used to detect pharmacokinetic parameters and mRNA expressions, respectively. Compared with the NS group, ZWD at medium- and high-dose groups could significantly induce CYP2C6 (*P* < 0.05) activity, while the mRNA expression (*P* < 0.05) increased only in the high-dose group. Additionally, CYP2C11 activity was induced and consistent with mRNA expression (*P* < 0.05). Moreover, ZWD could induce the activity of CYP3A1 (*P* < 0.05), but the mRNA expression showed no significant differences except in high-dose groups. Additionally, ZWD has no effects on CYP1A2, CYP2B1, CYP2C7, and CYP2D2. In conclusion, the significant inductive effects of ZWD on three CYP450 isoenzymes indicated that when ZWD was coadministrated with drugs mediated by these enzymes, not only should the potential herb-drug interactions (HDIs) be observed, but the dosage adjustment and tissue drug concentration should also be considered. Furthermore, the approach described in this article can be applied to study the importance of gender, age, and disease factors to HDI prediction.

## 1. Introduction

The Zhenwu decoction (ZWD), one of the classic prescriptions for the treatment of Yang deficiency, was recorded in the “Treatise on Febrile Diseases” by Zhang Zhongjing. It is composed of Aconiti Lateralis Radix Praeparata (the lateral radix of *Aconitum carmichaelii* Debx.), Zingiberis (rhizome of *Zingiber officinale* Rosc.), Atractylodis Macrocephalae Rhizoma (radix of *Atractylodes macrocephala* Koidz.), Paeoniae Radix Alba (radix of *Paeonia lactiflora* Pall.), and Poria (sclerotium of *Poria cocos* (Schw.) Wolf.), and modern researches have shown that ZWD has therapeutic effects on nephrotic syndrome [1], Parkinson's disease [2], and chronic heart failure [3]. Clinically, ZWD is often used in combination with other drugs [4]; however, this combination may be resulting in various herb-drug interactions (HDIs).

HDIs have recently attracted wide attention, and they will lead to changes of plasma drug concentration and further affect efficacy [5]. Metabolic interaction is particularly important in the HDI triggers, it is becoming the main pathway for clinical HDIs, accounting for approximately 40% [6], and cytochrome P450 (CYP450) oxidase plays a pivotal role in metabolic interactions [7]. The CYP450 enzyme is the most critical metabolic enzyme system involved in the biological transformation of exogenous and endogenous substances [8]. The vital enzymes of the CYP superfamily are CYP1, CYP2, and CYP3, while CYP1A2, CYP2B6, CYP2C8, CYP2C9, CYP2C19, CYP2D6, and CYP3A4 participated in 90% of clinical drug metabolism [9]. In rats, their orthologs are CYP1A2, CYP2B1, CYP2C7, CYP2C6, CYP2C11, CYP2D2, and CYP3A1, respectively [10–14].

So, with the growing demand for the safety assessment of clinical drugs, this presentation was focused on simultaneously elucidating the effects of ZWD on the activities and mRNA expressions of seven CYP450 isoenzymes in rats based on a validated UPLC-MS/MS method [5] and real-time quantitative reverse-transcription polymerase chain reaction (RT-qPCR) in order to promote the scientific and rational clinical use of ZWD.

## 2. Materials and Methods

### 2.1. Materials and Instruments

Higenamine (No. Q-078-150731) was purchased from Beijing Zhongke Quality Control Biotechnology, Inc. (Beijing, China). Paeoniflorin (No. 110736201539) and atractylenolide III (No. 73030-71-4) were obtained from the National Institutes for Food and Drug Control (Beijing, China). 6-Gingerol (No. MUST-17120205) was purchased from Chengdu Man Site Pharmaceutical Co., Ltd. (Chengdu, China). Dehydrotumulosic acid was purchased from Chengdu Chroma-Biotechnology Co., Ltd. (Chengdu, China). Probe drugs used include phenacetin, bupropion, diclofenac, amodiaquine, omeprazole, dextromethorphan, and midazolam (Nos. 100095-201205, 100671-200301, 100334-200302, 101217-201401, 100367-201305, 100201-201003, and 171250-202002, respectively). The standard materials were supplied by the Chinese Food and Drug Administration Research Institute (Beijing, China). Glibenclamide (internal standard, no. 171250) was purchased from Yuanye Biotechnology Co., Ltd. (Shanghai, China). The purity of the standards was higher than 98%. The following materials were also acquired: TRIzol Reagent (Invitrogen, Carlsbad, CA), Verso cDNA Synthesis Kit (Thermo Fisher Scientific, MA, USA), SYBR Green PCR Kit (Qiagen, Hilden, Germany), PCR primer (Moore Biotech, Hefei, China), and DEPC water (General Biotech, Shanghai, China).

The following instruments were used: Agilent 1290 Infinity (Agilent Technologies Inc., California, USA); AB SCIEX 4500 triple-quadrupole mass spectrometer (AB SCIEX, USA); real-time quantitative PCR (Applied Biosystems, CA, USA); Acquity CSH C_18_ Column (2.1 × 100 mm, 1.7 *μ*m, Waters Corp., Mass., USA); and Millipore Milli-Q purification system (Millipore, Bedford, USA).

### 2.2. Preparation for ZWD Extract and Probe Cocktail

The dried raw herbs of ZWD were purchased from Anhui Puren Chinese Herbal Medicine Co., Ltd. (Anhui, China); *Zingiber officinale* Roscoe was homemade (fresh Anhui-origin ginger, washed, and sliced). The extraction process was as follows: *Aconitum carmichaeli* Debx : *Poria cocos* (Schw.) Wolf : *Zingiber officinale* Roscoe : *Paeonia lactiflora* Pall : *Atractylodes macrocephala* Koidz (nos. 160401, 160506, 160521, and 160613, respectively) at the proportion of 9 : 9 : 9 : 9 : 6, soaked in 10 times distilled water for 30 min and then boiled for 1.5 h, filtered, and the residue boiled with 8 times water for 1 h again. The two filtrates were mixed and concentrated to 2.1 g/mL and stored at 4°C.

The proper amount of seven probe substrates were dissolved in a certain amount (about 1/3 to 1/2 of the total volume of normal saline (NS)) of NS and anhydrous ethanol (0.5 mL). Ultrasonically stirred until homogeneous, Tween 80 was slowly dropped into the solution until it was clear and transparent. Finally, the volume with NS was determined. Notably, the solution was prepared when we needed it, and the dose of mixed probe substrates was 1 mL/kg.

### 2.3. Characterization of ZWD Extract by HPLC

High-performance liquid chromatography (HPLC) was performed to support the stability and quality of the ZWD extract. The following chromatographic conditions were used: a Shimadzu LC-15C UV HPLC system with a C_18_ column (4.6 mm × 250 mm, 5 *μ*m), a column temperature of 30°C, a flow rate of 1 mL·min^−1^, a wavelength of 230 nm, and 0.05% phosphoric acid aqueous solution (A)-acetonitrile (B) gradient elution described as follows: 0-10 min, 91%-88% (A); 10-20 min, 88%-85% (A); 20-33 min, 85%-65% (A); 33-38 min, 65%-60% (A); 38-48 min, 60%-52% (A); 48-58 min, 52%-57% (A); and 58-62 min, 57%-91% (A).

### 2.4. Animal Treatment

All rats were kept at a room temperature of 20 ± 2°C with a 12 h light/dark cycle and 50 ± 5% relative humidity for one week. The use of animals reported here have been approved by the Institutional Animal Care and Use Committee of Anhui Medical University (Animal Medical Ethics Committee of Anhui Medical University LLSC20160336), and the experimental procedures were conducted in accordance with the Guidelines for Proper Conduct of Animal Experiments. 48 male Sprague-Dawley (SD) rats (240~280 g) were randomly divided into the NS group and the ZWD low- (2.1875 g/kg), medium- (4.375 g/kg), and high- (8.75 g/kg) dose groups (12 per group) [15]. After one week of adaptation under controlled temperature and humidity conditions, rats were given corresponding doses of ZWD or NS intragastrically once daily for 28 consecutive days. They were fasted, but water was provided ad libitum before the experiment.

### 2.5. Collection of Plasma and Liver Tissue Samples

On the 29th day, half of the 48 rats received a cocktail substrate solution through the tail vein at a dose of 1 mL/kg, and plasma samples were obtained following the established procedures [5]. Meanwhile, after the last administration of another 24 rats for 30 min, blood was taken from the abdominal aorta and the liver was rapidly separated. All samples were stored at -80°C for analysis.

### 2.6. Total RNA Isolation and cDNA Synthesis

Based on the manufacturer's protocol, total RNA was isolated from 50 mg liver samples using the TRIzol Reagent. Then, the RNA concentration was determined and the absorbance ratio (*A*_260_/*A*_280_) was in the range of 1.8-2.0, indicating the excellent quality of RNA [16]. The RNA pellet was stored at -80°C. According to the RevertAid First Strand cDNA Kit, total RNA (2 *μ*L) was reversely transcribed into cDNA at 42°C for 60 min, 70°C for 5 min, and 4°C for 5 min. Reverse-transcription products were stored at -80°C until analysis.

### 2.7. RT-qPCR Analysis

*β*-Actin as the housekeeping gene was selected. RT-qPCR was performed as follows: predenaturation at 95°C for 10 min, 95°C for 15 s, and 60°C for 60 s (40 cycles).

The forward and reverse primer sequences are listed in Table 1.

### 2.8. Statistical Analysis

The main pharmacokinetic parameters were calculated by noncompartmental analysis using the DAS 2.0 software, including area under the curve (AUC), half-life time (*T*_1/2_), clearance (CL), and volume (*V*). mRNA expressions were performed by 2^−ΔΔCT^ calculation. Results are expressed as mean ± standard deviation ($\bar{x}\pm \text{SD}$). The Kruskal-Wallis test, one-way of analysis of variance (ANOVA), and the least-significant difference (LSD) test were used for statistical parameter analysis. *P* < 0.05 was regarded as statistically significant.

## 3. Results

### 3.1. Characterization of ZWD Extract by HPLC

**Figure 1:**
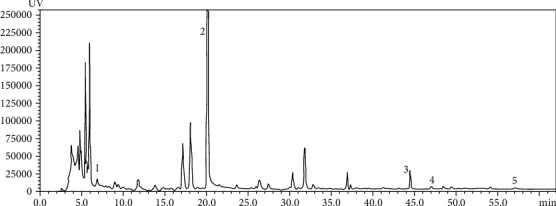
Identification of five components in ZWD by HPLC (peak 1: higenamine; peak 2: paeoniflorin; peak 3: atractylenolide III; peak 4: 6-gingerol; peak 5: dehydrotumulosic acid).

According to HPLC analysis, five compounds in ZWD extracts were identified as shown in Figure 1: higenamine (peak 1), paeoniflorin (peak 2), atractylenolide III (peak 3), 6-gingerol (peak 4), and dehydrotumulosic acid (peak 5), which provided evidence for the quality control of ZWD.

### 3.2. Validation of “Cocktail” Method

#### 3.2.1. Specificity and Linear Ranges

**Figure 2:**
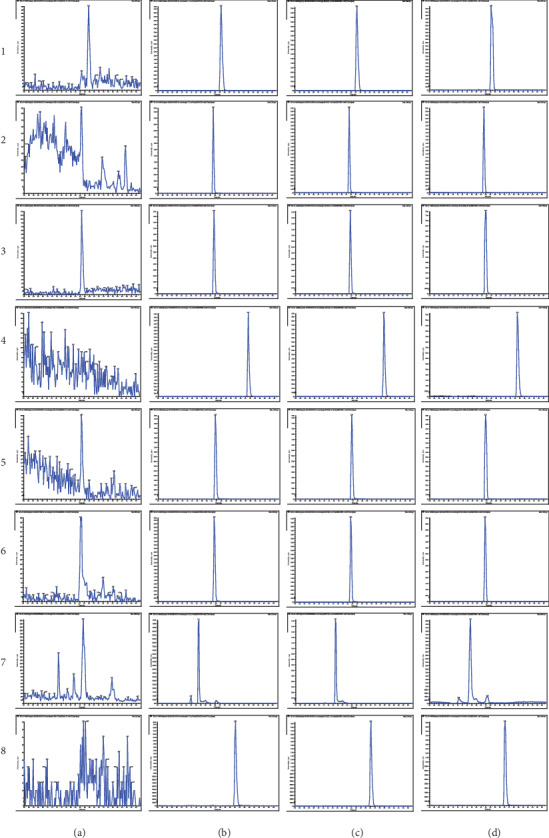
UPLC-MS/MS-specific chromatograms of seven probes and glibenclamide in rat plasma: (a) blank plasma; (b) probe substrates and glibenclamide (IS); (c) blank plasma spiked with phenacetin, bupropion, diclofenac, amodiaquine, omeprazole, dextromethorphan, midazolam, and IS (1-8); (d) plasma probe substrates and IS after receiving cocktail substrate solution through tail vein injection.

The specificity results (Figure 2) indicated that endogenous substances did not substantially interfere with the retention time of probe drugs and internal standard (IS) in blank plasma. The linear ranges of the seven probe substrates were 2-1400, 2.25-600, 30-9000, 0.6-100, 2-2000, 0.8-400, and 6-1800 (ng/mL), and the correction coefficients (*r*) were 0.9986, 0.9980, 0.9965, 0.9984, 0.9967, 09977, and 0.9996, respectively.

#### 3.2.2. Precision and Accuracy

Interday precision and intraday precision of the method were assessed by detecting the low limit of qualification (LLOQ) and the low-, medium-, and high-quantification concentrations (LQC, MQC, and HQC) of plasma samples. Relative standard deviation (RSD) values of the precision do not exceed ±15% (Tables 2 and 3).

#### 3.2.3. Matrix Effect

By comparing the different results of analytes added into the blank sample and ultrapure water, the matrix effect was determined at LQC, MQC, and HQC concentrations. The RSD values in Table 4 were less than 4%, indicating that the matrix effect of plasma is negligible for quantitative analysis of all samples.

#### 3.2.4. Stability

The stability of all probe drugs was evaluated by LQC and HQC samples under different experimental conditions, including short-term stability (4 h at room temperature (25°C), 8 h in the automatic sampler, and three freeze and thaw cycles, respectively) and long-term stability (7 d at -80°C). Results showed that the probe substrates tested were within the recommended limits, RSD < 10% (Table 5).

### 3.3. Effect of ZWD on Rat CYP1A2, CYP2B1, CYP2C7, and CYP2D2 Activities

**Figure 3:**
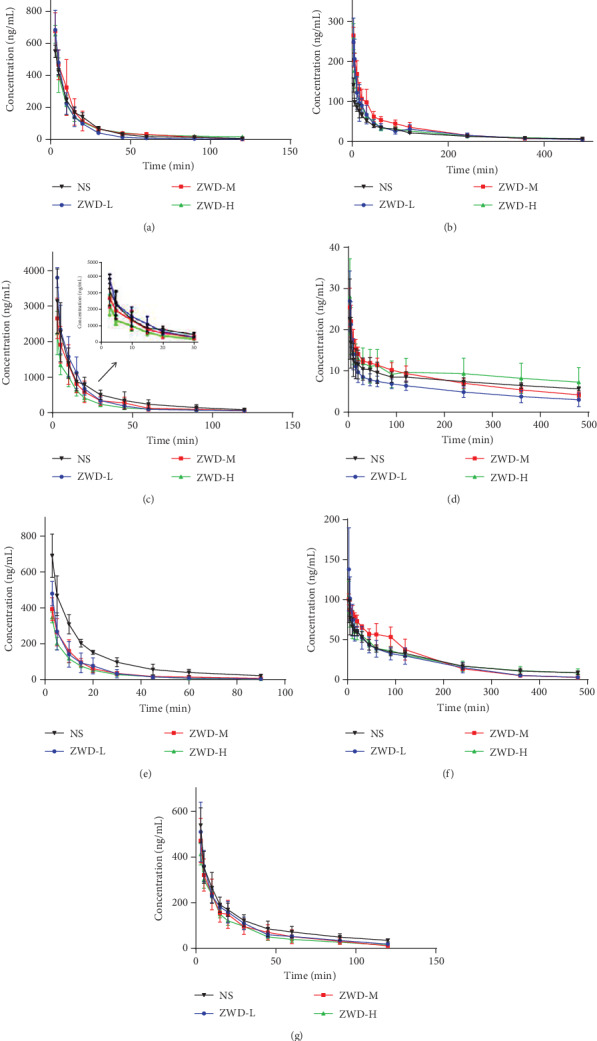
Mean concentration-time curves of seven probe substrates in rat plasma (ng/mL): (a) phenacetin, (b) bupropion, (c) diclofenac, (d) amodiaquine, (e) omeprazole, (f) dextromethorphan, and (g) midazolam.

The CYP1A2, CYP2B1, CYP2C7, and CYP2D2 activities were investigated by analyzing the pharmacokinetic parameters of phenacetin, bupropion, amodiaquine, and dextromethorphan, respectively.  Table 6 and Figure 3 present the main pharmacokinetic parameters and mean plasma concentration-time curves in different groups. Compared with the NS group, none of the differences measured were significant in ZWD groups (*P* > 0.05), except for *T*_1/2_ changes of bupropion, amodiaquine, and dextromethorphan (*P* < 0.05).

### 3.4. Effect of ZWD on Rat CYP2C6

To describe changes in CYP2C6 activity, Table 7 and Figure 3 present the pharmacokinetic profiles and mean plasma concentration-time curves of diclofenac. The results indicated that compared with the NS group, ZWD in medium and high dose groups significantly reduced AUC_0−*t*_ and AUC_0−∞_ (*P* < 0.05) in a dose-dependent manner, which was consistent with the CL and *V* values (*P* < 0.05), while no changes were observed between the ZWD-L and NS groups.

### 3.5. Effect of ZWD on Rat CYP2C11

Omeprazole is a specific probe substrate for rat CYP2C11. The corresponding pharmacokinetic profiles and the mean plasma concentration-time curves are presented in Table 8 and Figure 3, respectively. Plasma omeprazole AUC_0−*t*_ and AUC_0−∞_ were significantly reduced in a dose-dependent manner (*P* < 0.05), and the CL value was significantly increased (*P* < 0.05), which means that drug plasma concentration was decreased and drug metabolism was accelerated. Interestingly, the CL value was the lowest in the ZWD-M group, consistent with *T*_1/2_. Accordingly, ZWD affects the drug metabolism mediated by CYP2C11.

### 3.6. Effect of ZWD on Rat CYP3A1

In rats, midazolam was metabolized by CYP3A1, as shown in Table 9 and Figure 3. After oral administration, AUC_0−*t*_ and AUC_0−∞_ decreased and CL increased as the dose increased (*P* < 0.05); notably, *T*_1/2_ was also reduced significantly (*P* < 0.05) and *V* was approximately constant. Changes in pharmacokinetic parameters of midazolam in rats suggested that CYP2A1 enzyme activity was induced, leading to an acceleration of the metabolism as well as a reduction of the plasma drug concentration.

### 3.7. Effect of ZWD on the mRNA Expression of Seven CYPs

**Figure 4:**
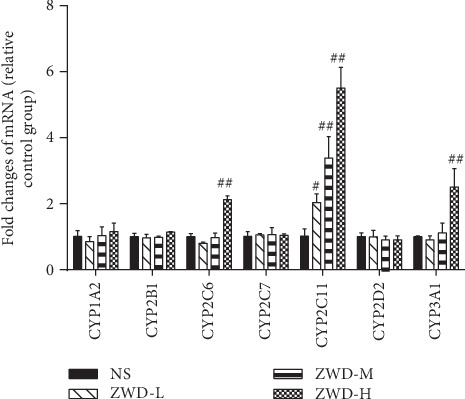
Effects of ZWD on fold changes of seven CYP450 isoenzyme mRNAs in rat liver compared with NS, ^#^*P* < 0.05 and ^##^*P* < 0.01.

As shown in Figure 4, compared with the NS group, the mRNA expressions of CYP2C6 and CYP3A1 only increased significantly to 2.08- and 2.50-fold in the ZWD-H dose group, respectively, while no significant difference was observed in the ZWD-L and ZWD-M dose groups. Moreover, CYP2C11 mRNA expression levels were significantly increased to 2.00-, 3.33-, and 5.41-fold in ZWD-L, ZWD-M, and ZWD-H dose groups, respectively. Being in line with the enzyme activity results, the mRNA expressions of CYP1A2, CYP2B1, CYP2C7, and CYP2D2 in the ZWD group were not significantly different from those in the NS group.

## 4. Discussion

Drug metabolism-induced interactions account for about half of HDIs, and CYP450 enzymes dominate in metabolism. At a basic level, a comprehensive assessment of the effects of ZWD on CYP450 enzymes is therefore critical for predicting HDIs during integrative medicine practice.

### 4.1. Selection of Rat Gender and ZWD Dose

CYP450 enzyme activity is influenced by species, gender, age, environment, medication, and pathological condition [17]. Similar to humans, since the CYP3A activity in male rats is 5 to 10 times higher than that in female rats, male rats were selected as experimental subjects [18, 19]. At the same time, this method is possible to elucidate different factors that affect the efficacy of ZWD.

For the dosage of ZWD, the *Formulaology* textbook of the National TCM Industry, “Ancient Classics List (First Batch)” and literature [20, 21] were referred to. According to the previous experimental results, the low, medium, and high doses of ZWD were 2.1875, 4.375, and 8.75 g/kg, respectively.

### 4.2. Explaination of ZWD Effects on CYP1A2, CYP2B1, CYP2C7, and CYP2D2

As mentioned in the literature, 82 volatile components were isolated by GC-MS, and five constituents of ZWD were qualified by HPLC [22, 23]. In these components, total glucoside of paeony has inductive effects on CYP1A2 and CYP2C9 enzyme activities [21]; ginger extract could inhibit the CYP2C19 and CYP1A2 enzyme activities [24, 25]; and Aconiti Lateralis Radix Praeparata has effects on various CYP450 enzyme activities, such as CYP3A, CYP2D, and CYP1A2 [26]. Interestingly, our study demonstrated that ZWD has no effect on CYP1A2, CYP2B1, CYP2C7, and CYP2D2. Basically, similar to the compatibility mechanism of the Siwu decoction, we hypothesized that due to the different effects of the ZWD components on metabolic enzymes, the interaction between them makes the integrated effects inconsistent with the individual contribution [27]. The results imply that the clinical use of ZWD is better than a single herb, showing the superior compatibility of Chinese medicine formulas.

### 4.3. Potential Mechanism of ZWD Effects on CYP2C6, CYP2C11, and CYP3A1

*In vivo*, CYP3A accounts for about 40% of the total liver P450 enzymes and mediates 50-60% clinical drug metabolism such as erythromycin, nimodipine, and lidocaine [28]. CYP2C occupies about 20% of the total P450 enzymes and mediates the hydroxylation metabolism reaction of losartan, toluene sulfonamide, and other drugs [29, 30]. Studies have shown that *Poria cocos* aqueous extract can significantly increase the CYP2C6, CYP2B1, and CYP3A1 activities and upregulate their mRNA expressions [5]. What's more, both *Poria cocos* and Aconiti Lateralis Radix can induce pregnane X receptor (PXR) activation and further mediate the transcription and expression of CYP3A4 [31, 32]. However, whether they induce CYP3A1 activity by activating PXR and showing synergy is unclear. Additional identification is needed. In addition, based on the above studies, the drug's regulation of enzyme activities at a stage independent of transcription, translation, and posttranslational protein modification may explain why high-dose ZWD induced CYP2C6 and CYP3A1 activities but upregulated mRNA expression. Taken together, the core finding of this experiment is that dose adjustment and HDI risk should be taken into consideration when ZWD is used with drugs metabolized by CYP2C6, CYP2C11, and CYP3A1. Meanwhile, *V* of diclofenac, omeprazole, and midazolam exceeded the total fluid volume, indicating that they are easily ingested into tissues. The concentration of drug in tissues, therefore, also needs to be monitored clinically in real time. Notably, *T*_1/2_ of midazolam was significantly decreased, CL was consistent with it, *V* is constant, and the metabolic process of midazolam conforms to the nonlinear pharmacokinetic model.

## 5. Conclusion

In summary, this dissertation was undertaken to evaluate the effects of ZWD on the activities and mRNA expressions of seven CYP450 enzymes by using a cocktail method. The method has proven to be sensitive, efficient, and reliable. Pharmacokinetic profile analysis shows that when ZWD is coadministrated with drugs metabolized by CYP2C6, CYP2C11, and CYP3A1, not only should the potential herb-drug interactions (HDIs) be observed but the dosage adjustment and tissue drug concentration should also be considered. Furthermore, the above approach can be applied to study the importance of gender, age, and disease factors to HDI prediction.

## Figures and Tables

**Table 1:** The primer for the enzymes and reference genes of rat.

CYP450	Forward primer	Reverse primer
CYP1A2	CATCTTTGGAGCTGGATTTG	CCATTCAGGAGGTGTCC
CYP2B1	AACCCTTGATGACCGCAGTAAA	TGTGGTACTCCAATAGGGACAAGATC
CYP2C6	TCAGCAGGAAAACGGATGTG	AATCGTGGTCAGGAATAAAAATAACTC
CYP2C7	TGTGAAGAACATCAGCCAATCCT	CACGGTCCTCAATGTTCCTTTT
CYP2C11	GGAGGAACTGAGGAAGAGCA	AATGGAGCATATCACATTGCAG
CYP2D2	GAAGGAGAGCTTTGGAGAGGA	AGAATTGGGATTGCGTTCAG
CYP3A1	TGCCAATCACGGACACAGA	ATCTCTTCCACTCCTCATCCTTAG
*β*-Actin	GCCCAGAGCAAGACAGGTAT	GGCCATCTCCTGCTCGAAGT

**Table 2:** Accuracy of seven probe substrates in rat plasma (*n* = 5).

Probe substrates	Mark concentration (ng/mL)	Accuracy, mean ± SD (ng/mL)	RSD (%)
Phenacetin	2	102.5 ± 7.79	7.59
	4	97.79 ± 5.19	5.31
	53	105.8 ± 5.05	4.77
	1050	107.1 ± 2.27	2.12

Bupropion	2.25	111.9 ± 8.38	7.49
	4.5	104.3 ± 5.89	5.65
	35	103.4 ± 2.76	2.67
	450	110.8 ± 3.48	3.14

Diclofenac	30	96.44 ± 8.85	9.17
	60	99.54 ± 4.12	4.14
	520	108.1 ± 6.25	5.78
	6750	96.09 ± 4.42	4.60

Amodiaquine	0.6	97.38 ± 10.9	11.2
	1.2	102.9 ± 8.42	8.18
	10	97.22 ± 6.40	6.58
	75	103.1 ± 4.99	4.84

Omeprazole	2	91.80 ± 7.01	7.63
	4	104.3 ± 6.63	6.36
	63.5	102.0 ± 5.36	5.25
	1500	103.0 ± 4.95	4.80

Dextromethorphan	0.8	106.1 ± 8.04	7.58
	1.6	100.9 ± 5.81	5.76
	17.9	106.5 ± 4.41	4.14
	300	98.17 ± 2.04	2.08

Midazolam	6	94.37 ± 7.13	7.56
	12	103.8 ± 6.44	6.20
	104	103.3 ± 4.18	4.04
	1350	98.52 ± 2.11	2.14

**Table 3:** Precision of seven probe substrates in rat plasma (*n* = 5).

Probe substrates	Added (ng/mL)	Interday		Intraday	
		Mean ± SD (ng/mL)	RSD (%)	Mean ± SD (ng/mL)	RSD (%)
Phenacetin	2	2.05 ± 0.16	7.59	2.07±0.13	6.49
	4	4.20 ± 0.22	5.14	4.28±0.29	6.84
	53	58.24 ± 2.21	3.79	55.91 ± 2.35	4.20
	1050	1125 ± 23.9	2.12	1086 ± 20.8	1.91

Bupropion	2.25	2.52 ± 0.19	7.49	2.47 ± 0.18	7.08
	4.5	4.70 ± 0.27	5.65	4.69 ± 0.24	5.15
	35	37.76 ± 0.72	1.90	37.09 ± 2.19	5.89
	450	447.6 ± 14.0	3.14	458.02 ± 6.38	1.39

Diclofenac	30	28.71 ± 1.15	4.00	27.96 ± 2.03	7.24
	60	55.47 ± 1.47	2.64	61.63 ± 2.63	4.28
	520	457.9 ± 13.4	2.93	475.1 ± 31.4	6.48
	6750	6091 ± 311.4	5.11	6381 ± 229.5	3.58

Amodiaquine	0.6	0.58 ± 0.07	11.8	0.61 ± 0.07	11.2
	1.2	1.24 ± 0.10	8.18	1.28 ± 0.09	7.15
	10	9.72 ± 0.64	6.58	10.35 ± 0.66	6.36
	75	77.35 ± 3.75	4.84	74.53 ± 3.25	4.35

Omeprazole	2	1.84 ± 0.14	7.63	1.90 ± 0.17	8.77
	4	4.17 ± 0.27	6.36	4.16 ± 0.32	7.72
	63.5	64.54 ± 3.39	5.25	63.46 ± 3.04	4.79
	1500	1545 ± 74.2	4.8	1533 ± 62.5	4.07

Dextromethorphan	0.8	0.87 ± 0.05	5.38	0.86 ± 0.05	6.03
	1.6	1.64 ± 0.12	7.09	1.71 ± 0.11	6.65
	17.9	18.64 ± 0.93	5.00	19.34 ± 1.01	5.25
	300	312.3 ± 6.63	2.12	314.2 ± 8.82	2.80

Midazolam	6	5.66 ± 0.43	7.56	5.82 ± 0.40	6.94
	12	12.46 ± 0.77	6.20	12.33 ± 0.76	6.18
	104	107.4 ± 4.34	4.04	107.2 ± 5.69	5.29
	1350	1330 ± 28.4	4.14	1382 ± 53.32	3.70

**Table 4:** Matrix effect of seven probe substrates in rat plasma (*n* = 6).

Mark concentration (ng/mL)	Compounds	Matrix effect (%)	RSD (%)
LQC	Phenacetin	96.04 ± 3.49	3.63
	Bupropion	95.50 ± 2.02	2.12
	Diclofenac	96.97 ± 3.24	3.35
	Amodiaquine	95.36 ± 3.22	3.38
	Omeprazole	96.35 ± 3.41	3.54
	Dextromethorphan	96.59 ± 2.08	2.15
	Midazolam	95.78 ± 3.14	3.28

MQC	Phenacetin	97.59 ± 2.30	2.35
	Bupropion	98.66 ± 1.74	1.77
	Diclofenac	98.09 ± 2.45	2.49
	Amodiaquine	95.80 ± 3.20	3.34
	Omeprazole	96.68 ± 1.09	1.12
	Dextromethorphan	97.90 ± 1.58	1.61
	Midazolam	95.81 ± 3.18	3.32

HQC	Phenacetin	94.79 ± 2.58	2.72
	Bupropion	97.21 ± 1.38	1.42
	Diclofenac	93.51 ± 2.07	2.22
	Amodiaquine	92.87 ± 3.48	3.75
	Omeprazole	96.00 ± 1.83	1.91
	Dextromethorphan	96.15 ± 1.19	1.23
	Midazolam	97.10 ± 2.57	2.65

**Table 5:** Stability of seven probe substrates in rat plasma (*n* = 5).

QC	Probe substrates	Stability (mean ± SD)			
		Room temperature	Automatic sampler	Multigelation	Long-term freeze
LQC	Phenacetin	4.12 ± 0.20	4.10 ± 0.07	4.12 ± 0.12	4.26 ± 0.15
	Bupropion	4.72 ± 0.31	4.77 ± 0.26	4.79 ± 0.29	4.76 ± 0.28
	Diclofenac	59.31 ± 2.72	59.16 ± 1.91	55.17 ± 3.72	63.06 ± 3.52
	Amodiaquine	1.24 ± 0.09	1.27 ± 0.10	1.25 ± 0.12	1.27 ± 0.08
	Omeprazole	4.11 ± 0.34	4.24 ± 0.29	4.07 ± 0.27	4.05 ± 0.38
	Dextromethorphan	1.74 ± 0.09	1.67 ± 0.15	1.65 ± 0.12	1.66 ± 0.14
	Midazolam	12.17 ± 0.88	12.20 ± 0.75	12.34 ± 0.94	11.85 ± 0.91

HQC	Phenacetin	1103 ± 29.9	1058 ± 12.9	1130 ± 14.4	1127 ± 15.7
	Bupropion	469.9 ± 14.9	461.7 ± 19.7	465.8 ± 10.9	475.1 ± 11.9
	Diclofenac	7611 ± 170.5	6673 ± 154.8	7061 ± 357.7	7072 ± 170.0
	Amodiaquine	75.02 ± 2.03	76.38 ± 2.35	81.19 ± 1.72	79.96 ± 2.97
	Omeprazole	1516 ± 70.0	1538 ± 77.4	1540 ± 59.3	1540 ± 76.4
	Dextromethorphan	297.9 ± 7.76	314.6 ± 16.0	320.7 ± 12.8	319.0 ± 11.9
	Midazolam	1310 ± 40.4	1413 ± 48.5	1339 ± 27.2	1370 ± 40.9

**Table 6:** Pharmacokinetic parameters of four probe substrates in rat plasma ($\bar{x}\pm \text{SD}$, *n* = 6).

	Parameter	NS	ZWD-L	ZWD-M	ZWD-H
Phenacetin	AUC_0−*t*_ (mg/L·min)	9.48 ± 0.57	10.49 ± 3.85	10.69 ± 2.48	10.11 ± 1.26
	AUC_0−∞_ (mg/L·min)	9.83 ± 0.77	10.59 ± 3.86	10.85 ± 2.47	11.25 ± 1.77
	*T*_1/2_ (min)	32.31 ± 12.8	24.03 ± 4.26	22.53 ± 7.60	49.92 ± 15.4
	CL (L/min/kg)	2.04 ± 0.16	2.06 ± 0.58	1.93 ± 0.44	1.82 ± 0.28
	*V* (L/kg)	93.76 ± 30.7	72.92 ± 26.4	75.67 ± 21.3	110.3 ± 18.2

Bupropion	AUC_0−*t*_ (mg/L·min)	11.10 ± 1.32	12.53 ± 3.25	13.38 ± 0.69	12.04 ± 1.03
	AUC_0−∞_ (mg/L·min)	13.03 ± 4.15	15.90 ± 7.27	15.76 ± 1.73	13.67 ± 1.90
	*T*_1/2_ (min)	78.27 ± 8.24	64.88 ± 13.8	55.69 ± 7.74^##^	71.34 ± 18.2
	CL (L/min/kg)	1.63 ± 0.37	1.48 ± 0.65	1.28 ± 0.13	1.49 ± 0.23
	*V* (L/kg)	485.2 ± 110.2	388.1 ± 248.8	320.2 ± 134.3	351.2 ± 43.9

Amodiaquine	AUC_0−*t*_ (mg/L·min)	3.76 ± 0.54	2.69 ± 0.59	3.79 ± 0.50	4.68 ± 1.74
	AUC_0−∞_ (mg/L·min)	8.92 ± 0.75	4.99 ± 3.39	5.96 ± 1.36	13.55 ± 8.45
	*T*_1/2_ (min)	236.4 ± 50.6	169.7 ± 118.9	126.9 ± 25.2^#^	245.2 ± 98.2
	CL (L/min/kg)	2.26 ± 0.20	5.28 ± 2.56	3.49 ± 0.69	2.50 ± 2.13
	*V* (L/kg)	2072 ± 385.5	2291 ± 488.9	1618 ± 443.7	1763 ± 596.4

Dextromethorphan	AUC_0−*t*_ (mg/L·min)	11.28 ± 1.71	10.08 ± 2.73	11.87 ± 1.37	11.37 ± 1.47
	AUC_0−∞_ (mg/L·min)	13.59 ± 3.58	10.60 ± 2.84	12.24 ± 1.26	14.71 ± 4.04
	*T*_1/2_ (min)	79.09 ± 15.67	46.69 ± 4.84^##^	40.29 ± 4.52^##^	72.01 ± 28.1
	CL (L/min/kg)	1.56 ± 0.39	2.05 ± 0.72	1.65 ± 0.18	1.46 ± 0.43
	*V* (L/kg)	397.2 ± 69.9	333.9 ± 135.7	244.5 ± 88.7	434.1 ± 130.5

Compared with NS, ^#^*P* < 0.05 and ^##^*P* < 0.01.

**Table 7:** Pharmacokinetic parameters of diclofenac in rat plasma ($\bar{x}\pm \text{SD}$, *n* = 6).

Parameter	NS	ZWD-L	ZWD-M	ZWD-H
AUC_0−*t*_ (mg/L·min)	61.96 ± 4.07	56.12±8.19	48.68 ± 13.1^#^	36.53 ± 5.89^##^
AUC_0−∞_ (mg/L·min)	67.46 ± 5.36	59.14 ± 8.67	51.11 ± 13.4^#^	41.01 ± 6.52^##^
*T*_1/2_ (min)	35.45 ± 7.81	41.07 ± 26.0	34.99 ± 9.03	52.61 ± 11.5
CL (L/min/kg)	0.32 ± 0.01	0.34 ± 0.05	0.41 ± 0.10^#^	0.50 ± 0.09^##^
*V* (L/kg)	15.58 ± 3.65	19.79 ± 10.6	21.28 ± 8.67	37.38 ± 7.39^##^

Compared with NS, ^#^*P* < 0.05 and ^##^*P* < 0.01.

**Table 8:** Pharmacokinetic parameters of omeprazole in rat plasma ($\bar{x}\pm \text{SD}$, *n* = 6).

Parameter	NS	ZWD-L	ZWD-M	ZWD-H
AUC_0−*t*_ (mg/L·min)	11.67 ± 1.34	5.86 ± 2.00^##^	5.62 ± 0.97^##^	4.47 ± 0.42^##^
AUC_0−∞_ (mg/L·min)	12.31 ± 1.59	5.94 ± 1.99^##^	5.85 ± 0.83^##^	4.55 ± 0.38^##^
*T*_1/2_ (min)	24.47 ± 8.51	20.55 ± 6.78	25.49 ± 11.1	17.57 ± 4.37
CL (L/min/kg)	1.65 ± 0.21	3.61 ± 0.87^##^	3.48 ± 0.46^##^	4.43 ± 0.41^##^
*V* (L/kg)	57.40 ± 19.4	112.4 ± 53.7	132.6 ± 67.2	114.0 ± 39.2

Compared with NS, ^#^*P* < 0.05 and ^##^*P* < 0.01.

**Table 9:** Pharmacokinetic parameters of midazolam in rat plasma ($\bar{x}\pm \text{SD}$, *n* = 6).

Parameter	NS	ZWD-L	ZWD-M	ZWD-H
AUC_0−*t*_ (mg/L·min)	13.55 ± 2.56	11.57 ± 1.39	10.77 ± 2.98^#^	9.50 ± 0.96^##^
AUC_0−∞_ (mg/L·min)	16.08 ± 2.92	12.61 ± 1.30^#^	11.34 ± 2.95^##^	10.27 ± 1.07^##^
*T*_1/2_ (min)	53.32 ± 14.4	38.15 ± 9.11^#^	31.88 ± 9.42^##^	34.45 ± 5.92^##^
CL (L/min/kg)	1.28 ± 0.22	1.60 ± 0.17	1.88 ± 0.54^#^	1.97 ± 0.22^##^
*V* (L/kg)	97.36 ± 26.3	88.59 ± 24.8	90.43 ± 47.8	97.26 ± 15.5

Compared with NS, ^#^*P* < 0.05 and ^##^*P* < 0.01.

## Data Availability

The XLSX data used to support the findings of this study have not been made available because some interesting new experimental results that need further study have been found, and the data need to be protected temporarily.

## Abbreviations

ZWD: Zhenwu decoction
CYP450: Cytochrome P450 enzyme
HDI: Herb-drug interaction
UPLC-MS/MS: Ultraperformance liquid chromatography/tandem mass spectrometry
HPLC: High-performance liquid chromatography
RT-qPCR: Real-time quantitative reverse-transcription polymerase chain reaction
NS: Normal saline
AUC: Area under the curve
*T*_1/2_: Half-life time
CL: Clearance
*V*: Volume
IS: Internal standard
LLOQ: Low limit of qualification
LQC, MQC, and HQC: Low-, medium-, and high-quantification concentration
RSD: Relative standard deviation
PXR: Pregnane X receptor.
